# Supplementary material for: Exploring Competitive Relationship Between Haemophilus parainfluenzae and Mitis Streptococci via Co-Culture-Based Molecular Diagnosis and Metabolomic Assay
Source: Microorganisms. 2025 Jan 26;13(2):279. doi: 10.3390/microorganisms13020279 (PMC11857835; doi:10.3390/microorganisms13020279)
Supplement: Supplementary file 1 [file microorganisms-13-00279-s001.zip › Supplementary Figure S1.pdf]

A *Streptococcus mitis* _ in silico test

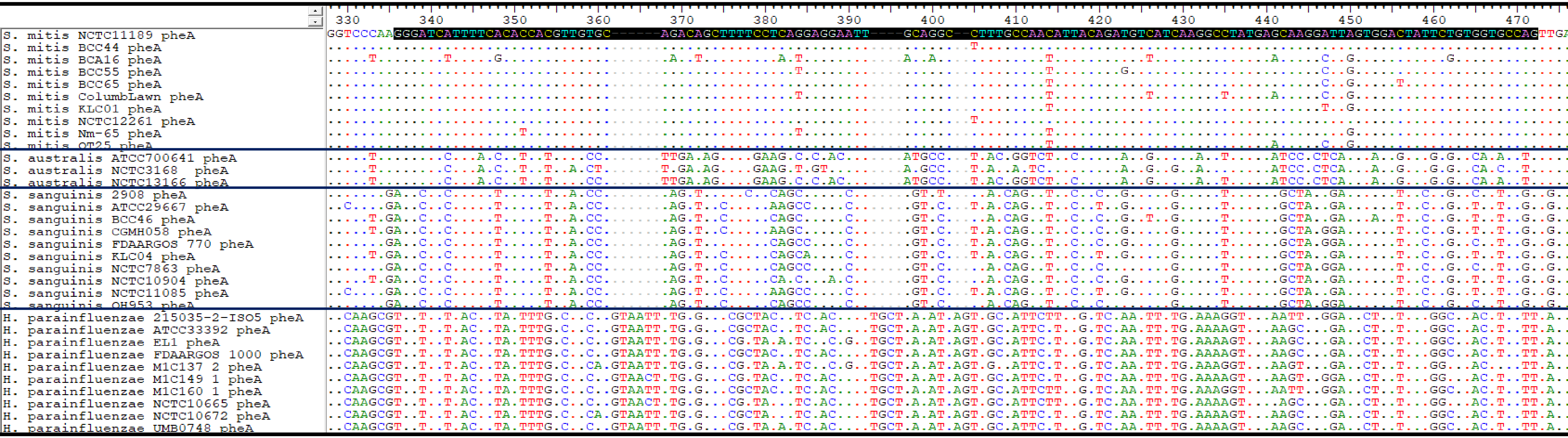

Descriptions | Graphic Summary | Alignments | **Taxonomy**

Reports | **Lineage** | Organism | Taxonomy

100 sequences selected

Organism	Blast Name	Score	Number of Hits	Description
Streptococcus	firmicutes		100	
. Streptococcus mitis	firmicutes	226	5	Streptococcus mitis hits
. Streptococcus mitis NCTC 12261	firmicutes	226	1	Streptococcus mitis NCTC 12261 hits
. Streptococcus toyakuensis	firmicutes	220	1	Streptococcus toyakuensis hits
. Streptococcus pneumoniae	firmicutes	215	89	Streptococcus pneumoniae hits
. Streptococcus gwangjuense	firmicutes	215	1	Streptococcus gwangjuense hits
. Streptococcus parapneumoniae	firmicutes	215	1	Streptococcus parapneumoniae hits
. Streptococcus pneumoniae ST556	firmicutes	215	1	Streptococcus pneumoniae ST556 hits
. Streptococcus pneumoniae A026	firmicutes	215	1	Streptococcus pneumoniae A026 hits

B *Streptococcus australis* _ in silico test

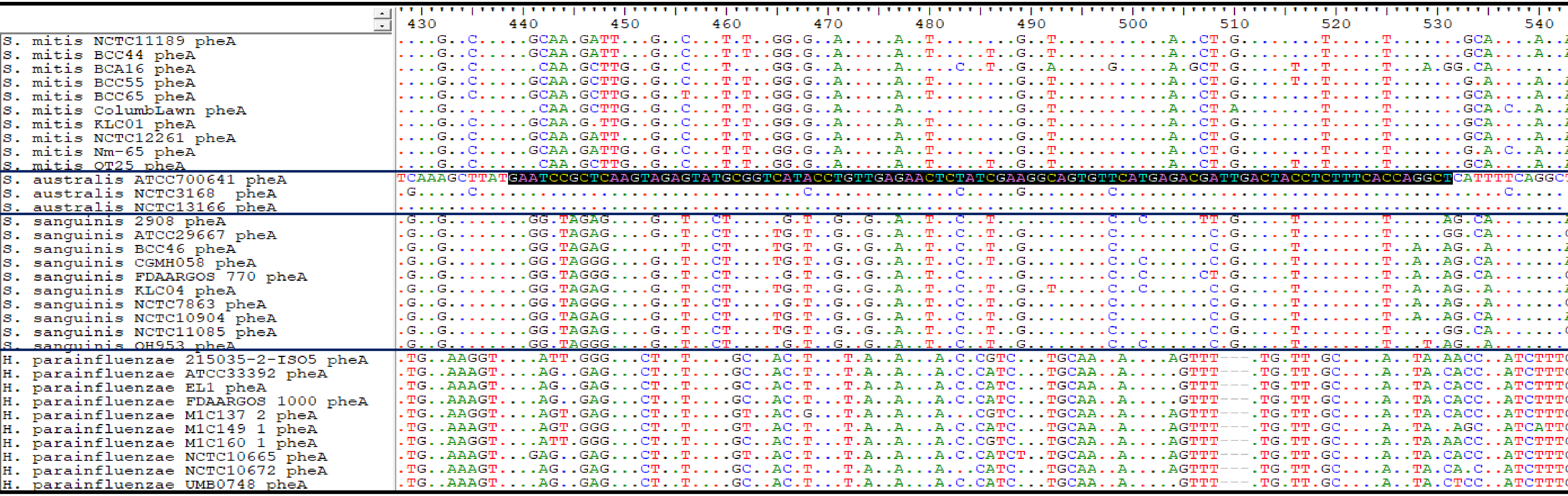

Descriptions | Graphic Summary | Alignments | **Taxonomy**

Reports | **Lineage** | Organism | Taxonomy

5 sequences selected

Organism	Blast Name	Score	Number of Hits	Description
Streptococcus	firmicutes		5	
. Streptococcus australis	firmicutes	172	2	Streptococcus australis hits
. Streptococcus viridans	firmicutes	150	1	Streptococcus viridans hits
. Streptococcus sp. A12	firmicutes	128	1	Streptococcus sp. A12 hits
. Streptococcus rubneri	firmicutes	128	1	Streptococcus rubneri hits

C *Streptococcus sanguinis* _ in silico test

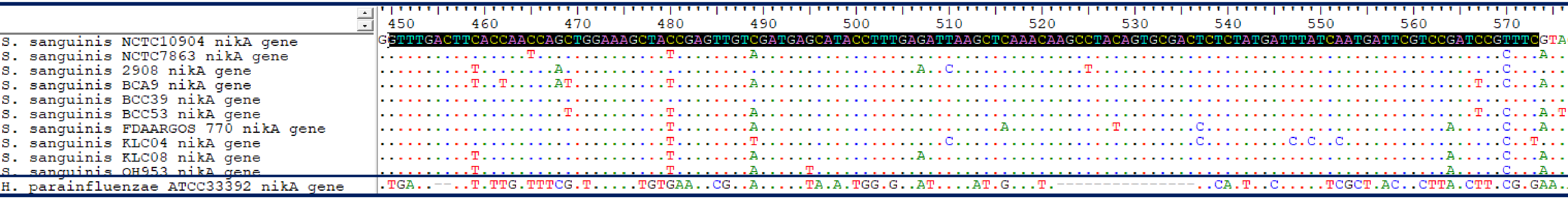

Descriptions | Graphic Summary | Alignments | **Taxonomy**

Reports | **Lineage** | Organism | Taxonomy

7 sequences selected

Organism	Blast Name	Score	Number of Hits	Description
Streptococcus	firmicutes		7	
. Streptococcus sanguinis	firmicutes	230	5	Streptococcus sanguinis hits
. Streptococcus sanguinis SK36	firmicutes	207	1	Streptococcus sanguinis SK36 h
. Streptococcus sp. DTU_2020_1000888_1_SI_GRL_NUU_041A	firmicutes	196	1	Streptococcus sp. DTU_2020_1(

D *Haemophilus parainfluenzae*_ in silico test

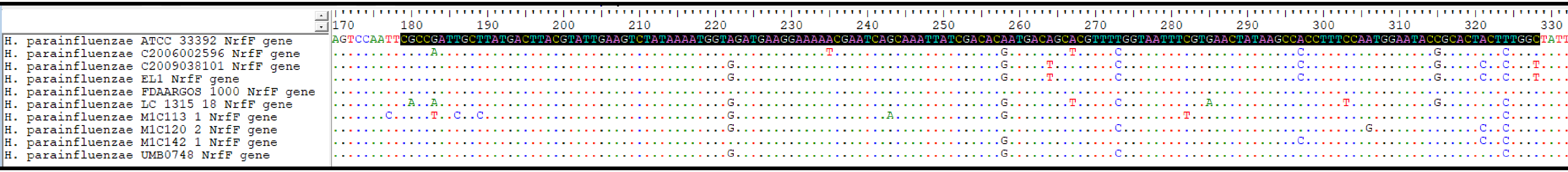

Descriptions | Graphic Summary | Alignments | **Taxonomy**

Reports | **Lineage** | **Organism** | **Taxonomy**

19 sequences selected

Organism	Blast Name	Score	Number of Hits	Description
Haemophilus	g-proteobacteria		19	
. Haemophilus parainfluenzae	g-proteobacteria		17	
. . Haemophilus parainfluenzae ATCC 33392	g-proteobacteria	278	1	Haemophilus parainfluenzae ATCC 33
. . Haemophilus parainfluenzae T3T1	g-proteobacteria	239	1	Haemophilus parainfluenzae T3T1 hits
. Haemophilus parainfluenzae	g-proteobacteria	278	17	Haemophilus parainfluenzae hits

E Species-specific primer PCR test result

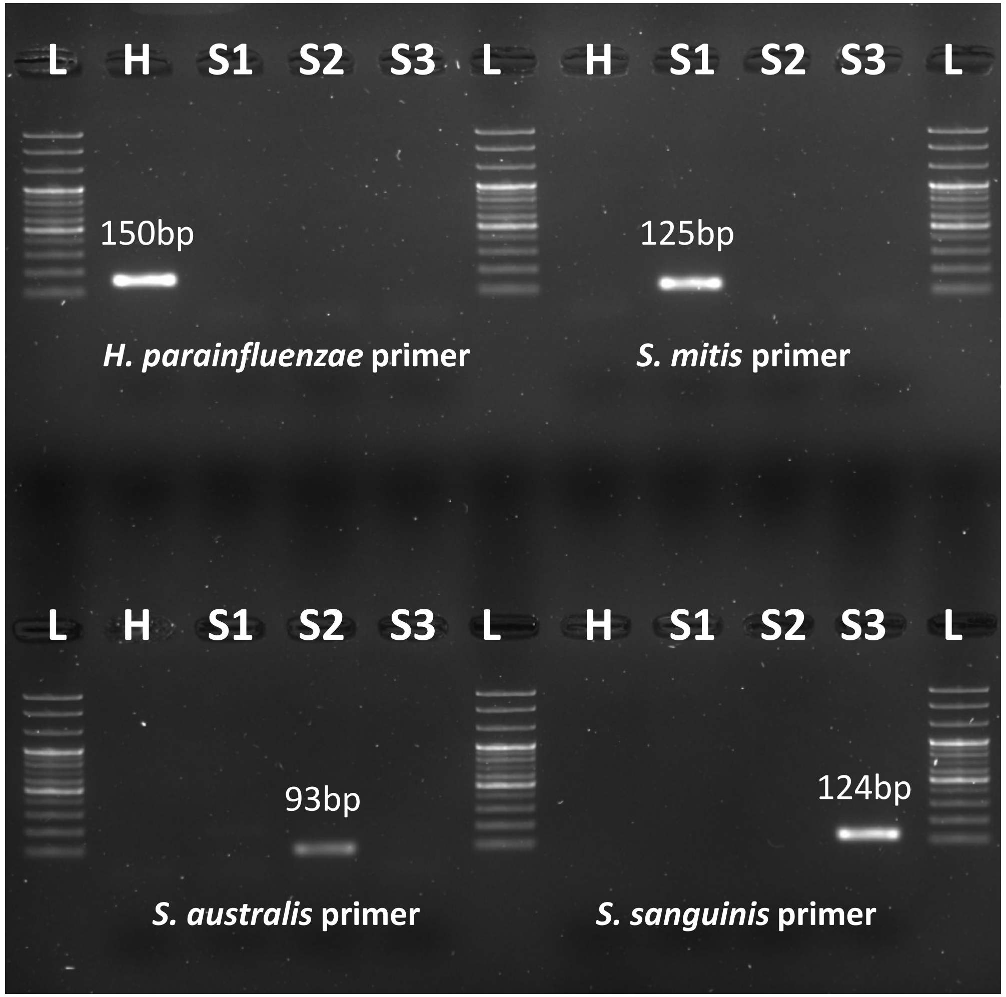

L : 100bp Ladder

H : *H. parainfluenzae*

S1 : *S. mitis* / S2 : *S. australis* / S3 : *S. sanguinis*

	Primer	Sequence	GC %	Tm
H	Foward	CGCCGATTGCTTATGACTTACG	50	62.1
	Reverse	GCCAAAGTAGTGCGGTATTCC	52	61.2
S1	Foward	GGGATCATTTTCACACCACGTTG	48	62.9
	Reverse	CTGGCACCACAGAATAGTCC	55	60
S2	Foward	GAATCCGCTCAAGTAGAGTATGC	48	62.9
	Reverse	AGCCTGGTGAAAGAGGTAGTC	55	62.1
S3	Foward	GTTTGACTTCACCAACCAGCTGG	52	64.6
	Reverse	GAAGCGGATCGGACGAATCATTG	52	64.6
