# Supplementary material for: Exploring Competitive Relationship Between Haemophilus parainfluenzae and Mitis Streptococci via Co-Culture-Based Molecular Diagnosis and Metabolomic Assay
Source: Microorganisms. 2025 Jan 26;13(2):279. doi: 10.3390/microorganisms13020279 (PMC11857835; doi:10.3390/microorganisms13020279)
Supplement: Supplementary file 1 [file microorganisms-13-00279-s001.zip › Supplementary Figure S2.pdf]

Nitrate reduction test_monoculture data

Nitrite detection

1. Treat Reagent A+B

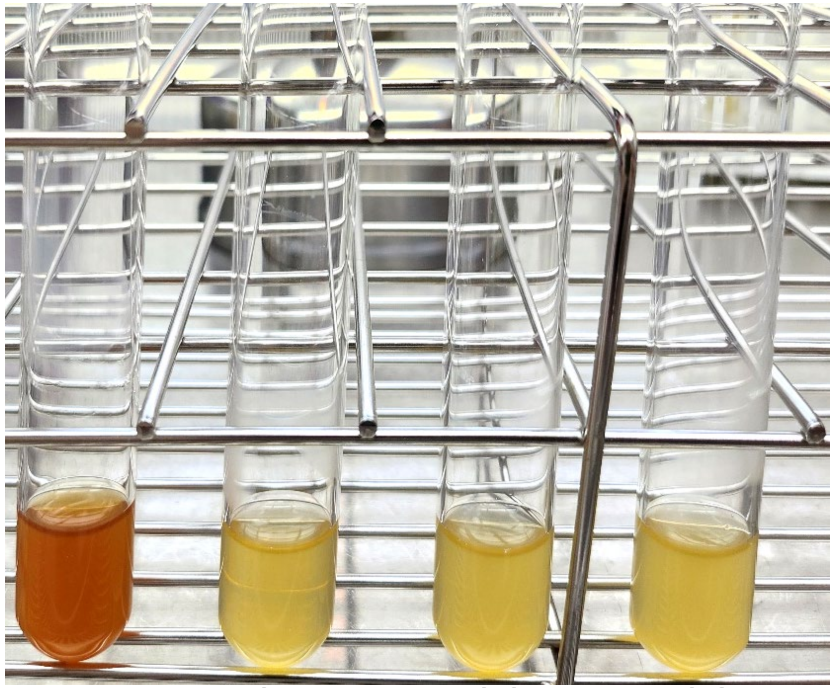

H S1 S2 S3

Nitrate detection

1. Treat Zinc dust

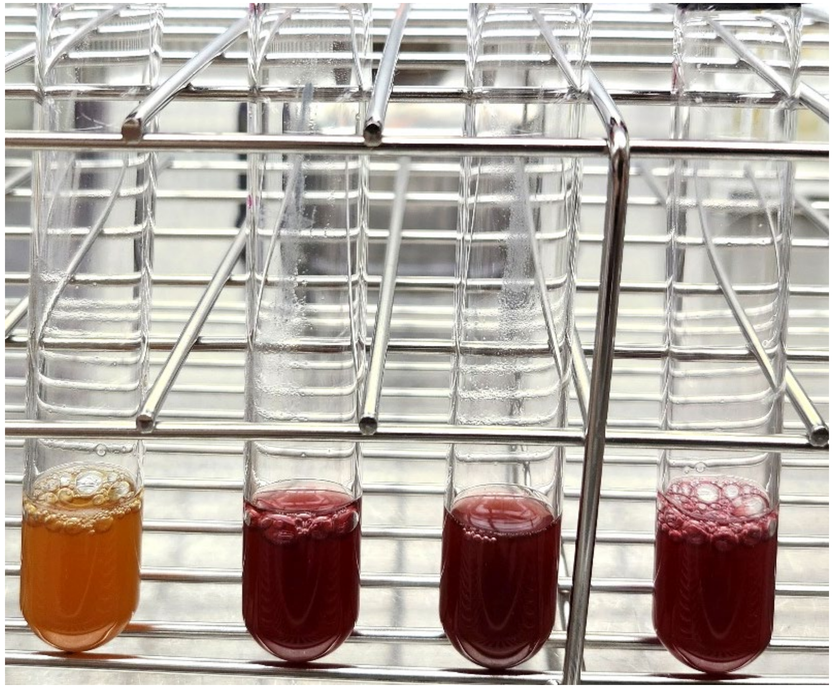

H S1 S2 S3

H: *Haemophilus parainfluenzae*
S1: *Streptococcus mitis*
S2: *Streptococcus australis*
S3: *Streptococcus sanguinis*
