# Supplementary material for: Exploring Competitive Relationship Between Haemophilus parainfluenzae and Mitis Streptococci via Co-Culture-Based Molecular Diagnosis and Metabolomic Assay
Source: Microorganisms. 2025 Jan 26;13(2):279. doi: 10.3390/microorganisms13020279 (PMC11857835; doi:10.3390/microorganisms13020279)
Supplement: Supplementary file 1 [file microorganisms-13-00279-s001.zip › Supplementary Figure S3.pdf]

Positive control metabolite venn diagram

NC: Nitrate non-treated group

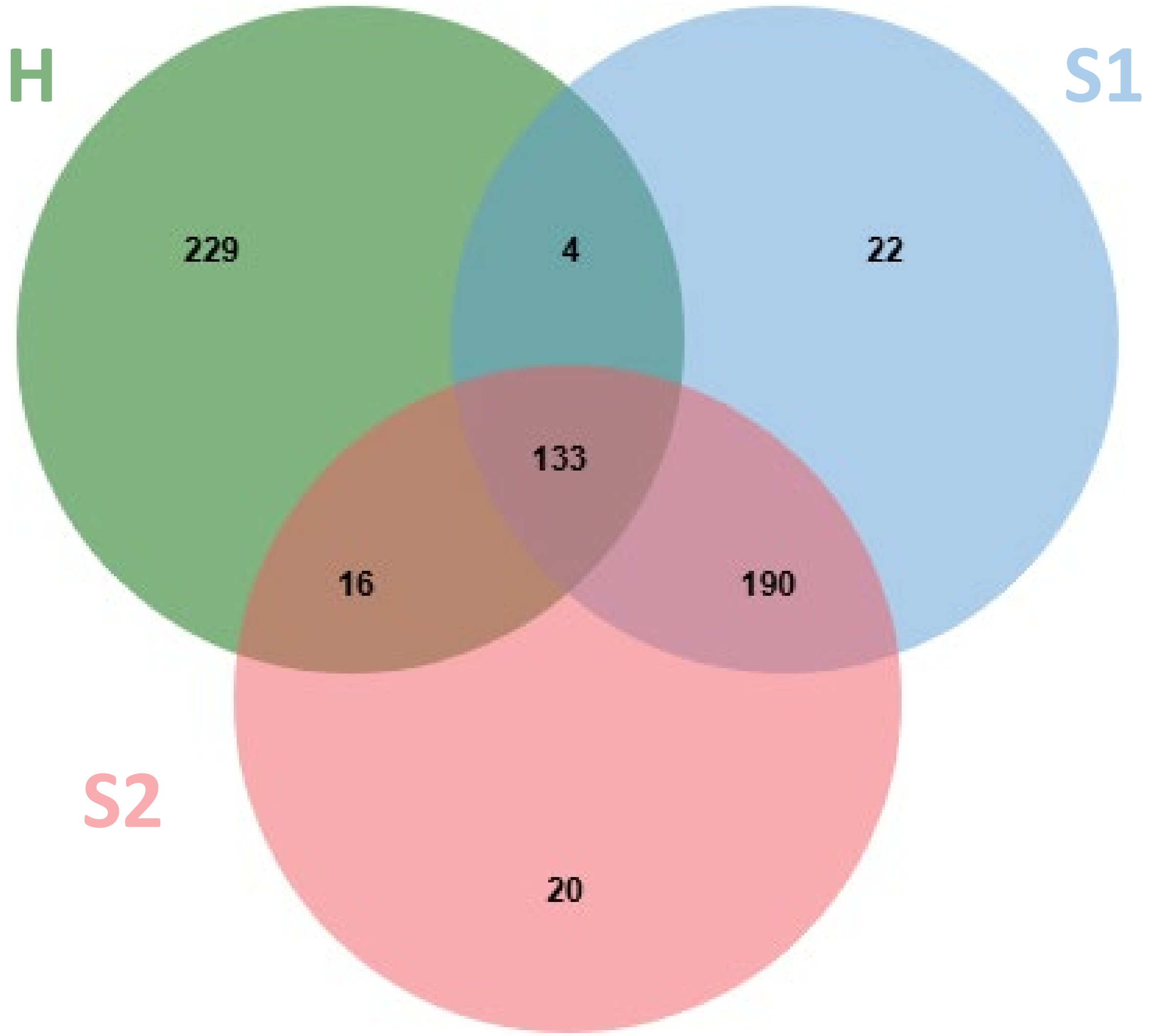

Case: Nitrate treated group

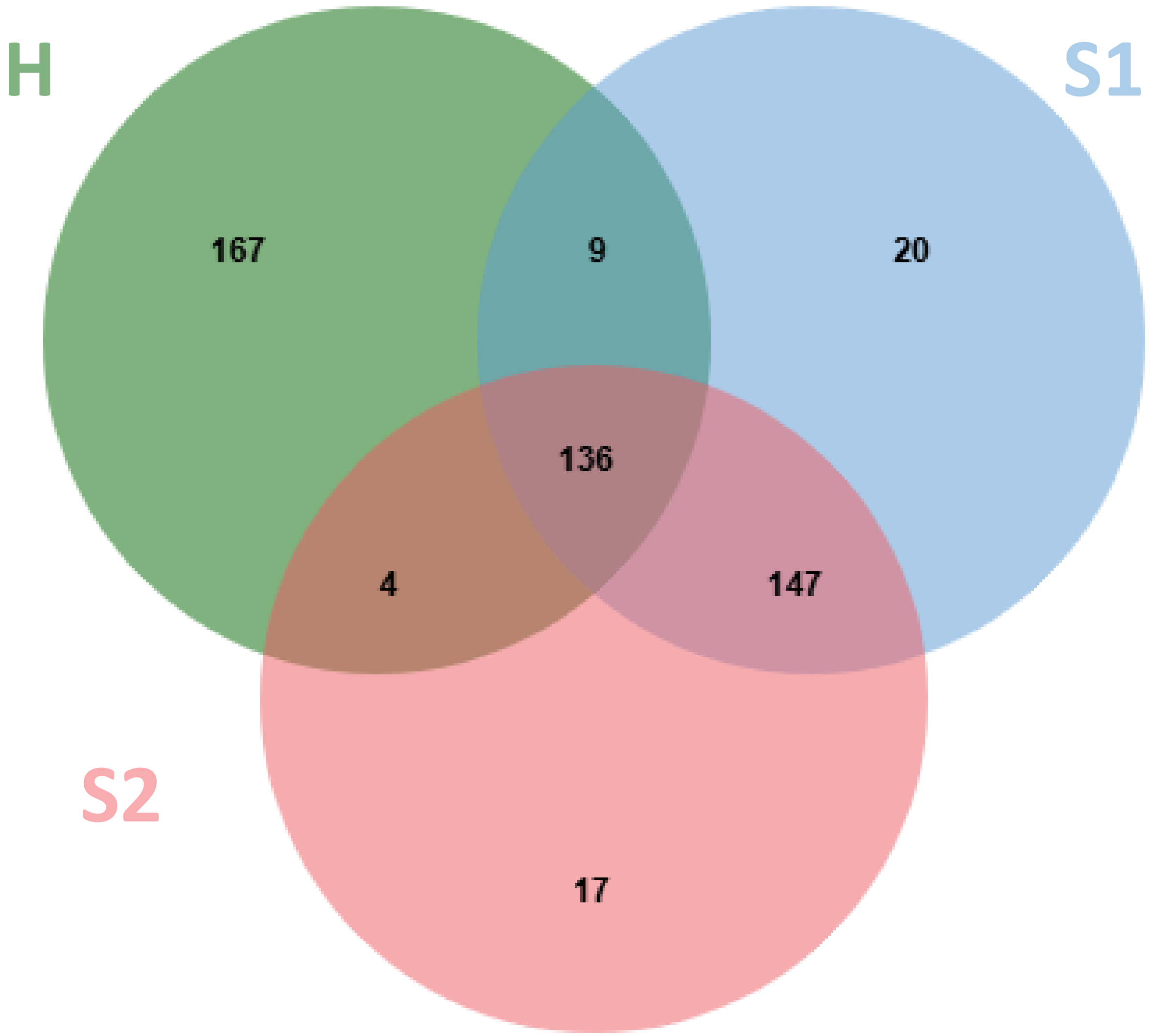

H: *H. parainfluenzae*; S1: *S. australis*; S2: *S. sanguinis*
