# Supplementary material for: Exploring Competitive Relationship Between Haemophilus parainfluenzae and Mitis Streptococci via Co-Culture-Based Molecular Diagnosis and Metabolomic Assay
Source: Microorganisms. 2025 Jan 26;13(2):279. doi: 10.3390/microorganisms13020279 (PMC11857835; doi:10.3390/microorganisms13020279)

*$Log_2(FC)$: Metabolite concentrations compared between cultivation times ($Log_2(FC)>2$, p value<0.05)

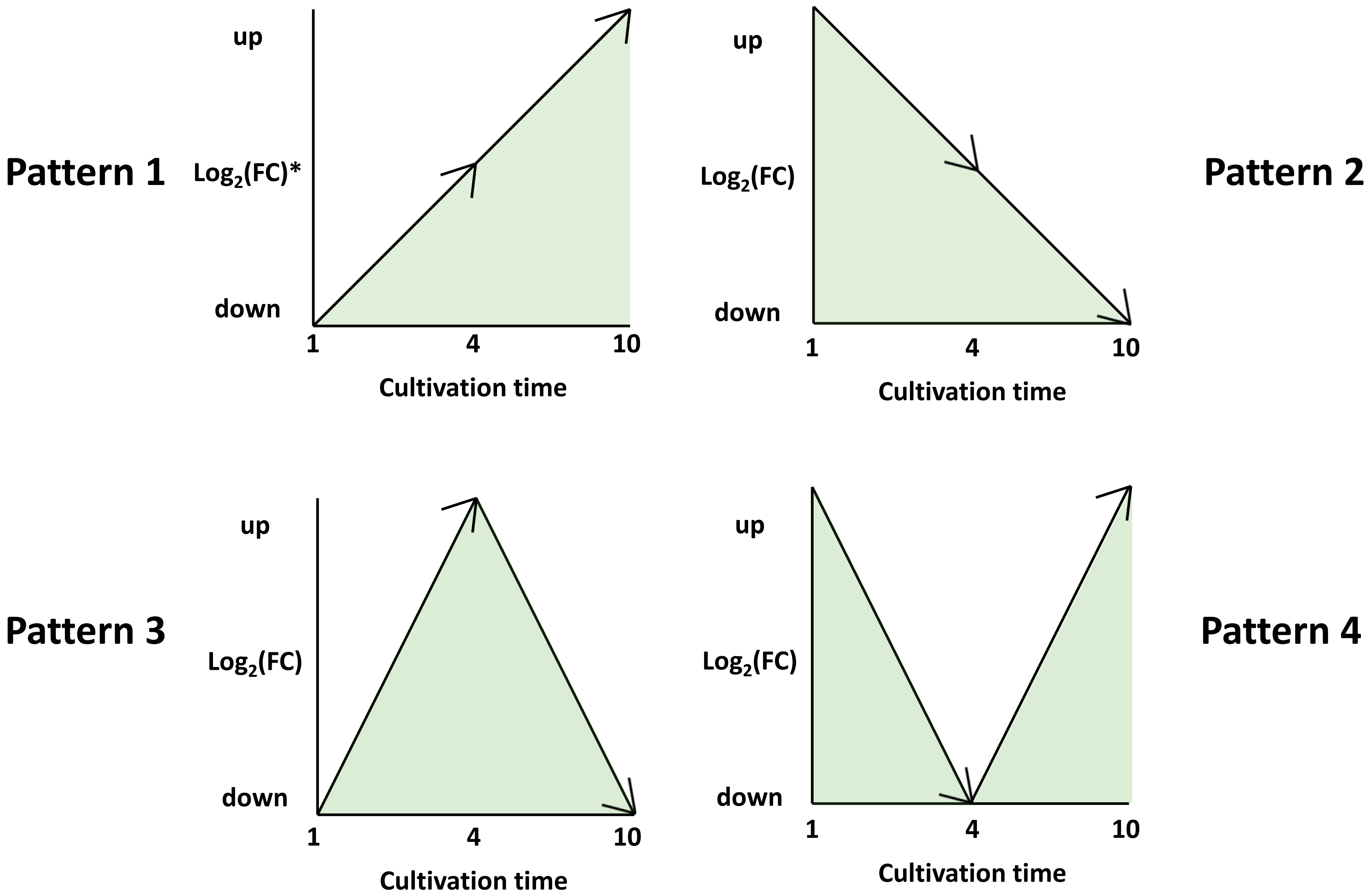

Supplement: Supplementary file 1 [file microorganisms-13-00279-s001.zip › Supplementary Figure S4.pdf]
